# Unified access to up-to-date residue-level annotations from UniProtKB and other biological databases for PDB data

**DOI:** 10.1038/s41597-023-02101-6

**Published:** 2023-04-12

**Authors:** Preeti Choudhary, Stephen Anyango, John Berrisford, James Tolchard, Mihaly Varadi, Sameer Velankar

**Affiliations:** 1 Protein Data Bank in Europe, European Molecular Biology Laboratory, European Bioinformatics Institute (EMBL-EBI), Wellcome Genome Campus, Hinxton, Cambridge, CB10 1SD UK; 2 AstraZeneca, Biomedical Campus, 1 Francis Crick Ave, Trumpington, Cambridge, CB2 0AA UK; 3 Claude Bernard University, Villeurbanne, Lyon, 69100 France

**Keywords:** Data integration, Protein databases, Data publication and archiving

## Abstract

More than 61,000 proteins have up-to-date correspondence between their amino acid sequence (UniProtKB) and their 3D structures (PDB), enabled by the Structure Integration with Function, Taxonomy and Sequences (SIFTS) resource. SIFTS incorporates residue-level annotations from many other biological resources. SIFTS data is available in various formats like XML, CSV and TSV format or also accessible via the PDBe REST API but always maintained separately from the structure data (PDBx/mmCIF file) in the PDB archive. Here, we extended the wwPDB PDBx/mmCIF data dictionary with additional categories to accommodate SIFTS data and added the UniProtKB, Pfam, SCOP2, and CATH residue-level annotations directly into the PDBx/mmCIF files from the PDB archive. With the integrated UniProtKB annotations, these files now provide consistent numbering of residues in different PDB entries allowing easy comparison of structure models. The extended dictionary yields a more consistent, standardised metadata description without altering the core PDB information. This development enables up-to-date cross-reference information at the residue level resulting in better data interoperability, supporting improved data analysis and visualisation.

## Introduction

As of March 2023, the Protein Data Bank (PDB)^1^ contains over 200,000 entries representing over 61,000 unique entries in the Universal Protein Resource Knowledgebase (UniProtKB)^2^. Often, the PDB archive has the same protein in multiple entries under different experimental conditions or interacting with different macromolecules (proteins, DNA, RNA) or ligand molecules^3–5^. Multiple 3-dimensional coordinates of the same protein are invaluable for comparative structure-function studies^3,6,7^. Linking structure data with annotations available in other data resources such as UniProtKB^2^ and to the structural and functional annotations is critical in order to understand biological function and processes at a molecular level. However, one of the barriers to comparative analysis or data integration is the independent, depositor-provided residue numbering in the coordinate files, which may not be the same as the protein sequence numbering^8^. While solving a protein 3D structure, many times the experiments are carried out only on a part of complete protein molecules (e.g. a domain) to make the sample amenable to experimental methods, especially in cases where there are highly flexible linker regions or intrinsically disordered regions^9,10^. Around 58% of the structures in the PDB contain smaller fragments (e.g. a domain) corresponding to different regions of a protein sequence. To determine where these fragments are located on the full-length protein sequence, these fragments need to be mapped to a common reference e.g. protein sequence numbering from a relevant entry in the UniProtKB database. The situation becomes complicated as often the flexible regions in the protein molecules are not modelled leading to unobserved residues i.e. residues without atomic coordinates in protein structures. The occurrence of missing residues makes structure-to-sequence mapping even more challenging. To address this fundamental problem of standardising residue numbering to make protein structure data more accessible to the broader scientific community, the PDBe^11^ and UniProtKB^2^ teams collaborated to establish the Structure Integration with Function, Taxonomy and Sequences (SIFTS) resource in 2002^12,13^. SIFTS provides up-to-date residue level mapping, with each weekly PDB release, between UniProtKB protein sequences and PDB protein structures allowing better integration of annotations based on protein sequence and structure.

In addition to mapping PDB structures to UniProtKB sequences, SIFTS also maps to other biological resources such as Pfam^14^, InterPro^15^, SCOP^16^, CATH^17^, IntEnz^18^, GO^19,20^, Ensembl^21^, NCBI taxonomy database^22^ and Homologene^23^.

In the past 20 years, SIFTS has become an essential resource, and its data provides the foundation of many data services and web pages. SIFTS is fundamental to the PDBe and PDBe-KB data resources^24^ and other databases, such as UniProtKB^2^, Pfam^14^, RCSB PDB^25^, PDBj^26^, SCOP2^27^, InterPro^15^ and MobiDB^28^, rely on SIFTS to fetch cross-references between PDB structures and other biological databases. SIFTS data is distributed as summary flat files in CSV/TSV formats and also as a detailed per-entry XML files with residue-level information available from the EMBL-EBI FTP area (ftp://ftp.ebi.ac.uk/pub/databases/msd/sifts/). SIFTS data is also accessible via the PDBe API^29^.

While SIFTS data has significantly improved the interoperability of PDB structure data with other key data resources, it still requires to be accessed separately from the 3D coordinates data in the PDB. The SIFTS output format is incompatible with 3D visualisation software that use the PDBx/mmCIF standard^30^ and requires an additional step of parsing the data to display SIFTS annotations on protein 3D structure. To boost the FAIRness^31^ (Findable, Accessible, Interoperable and Reusable) by further improving the findability and interoperability of PDB structures, the next logical step is to integrate SIFTS annotations alongside the 3D coordinates in the PDBx/mmCIF files. Moreover, with the availability of numerous high-quality, predicted protein structure models from resources like SWISS-MODEL^32^ and AlphaFold DB^33,34^, which generally follow the protein sequence numbering scheme, it was timely and essential to augment the protein sequence numbering for the experimentally determined 3D coordinates in the PDB. Using data from SIFTS resource, the PDBrenum^8^ web server replaces author sequence numbering with UniProtKB numbering in PDB or PDBx/mmCIF format files but it has certain limitations while handling special cases. For instance, while renumbering if this web server does not find any mapping data in SIFTS, it simply adds a large number to the residue’s sequence position number. These residues can be expression tags or insertions and need to be represented appropriately without losing the experimental context of the sample. Similarly, for chimeric proteins which are mapped to more than one protein sequence (UniProtKB accession), PDBrenum only renumbers according to the one protein sequence which has maximum coverage, losing information about remaining proteins in the chimeric construct. It does not integrate annotations to other data resources from SIFTS like Pfam, SCOP2 and CATH as well. Thus, there is a need to find a more consistent, sustainable and up-to-date solution while incorporating UniProtKB numbering and annotations from various other data resources in the 3D coordinate files.

Here, we describe incorporating SIFTS annotations in extended PDBx/mmCIF files to directly incorporate UniProtKB residue numbering next to the atomic coordinates. The PDBx/mmCIF is an extensible format that also provides a mechanism to maintain data integrity and is the master format for macromolecular structure data in the PDB^35^. We describe how this current work extends the PDBx/mmCIF dictionary by leveraging the extensibility of its structured framework, thereby providing a mechanism to enrich the biological context of a PDB structure.

## Results

### Extension to the core SIFTS pipeline

**Fig. 1:**
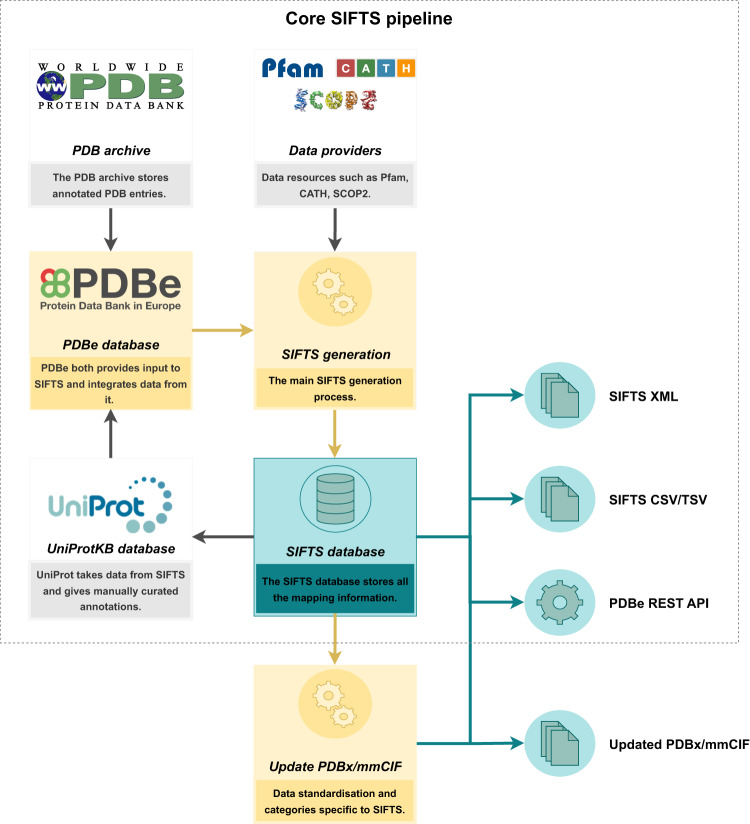
The schematic overview of the core SIFTS pipeline and an additional process for exporting data into PDBx/mmCIF Files. The figure illustrates the different components of the core SIFTS pipeline, represented in yellow, and the corresponding outputs, indicated in green. The core SIFTS process generates various output files, including the SIFTS database, XML, CSV, and TSV files. The additional process, represented in the figure, is responsible for augmenting SIFTS data in updated PDBx/mmCIF files. The grey components in the figure denote data resources that are external to the SIFTS pipeline.

The core SIFTS pipeline^13^ includes (1) a semi-automated process to retrieve the manually curated UniProtKB cross-reference (or canonical UniProtKB accession) for each protein chain in the PDB and (2) an automated process that generates residue-level correspondences between structure (PDB) and the corresponding sequence (UniProtKB). Initial mapping of UniProtKB sequence to the PDB structure is manually curated during the wwPDB annotation process^36^. During the semi-automated process, these manually curated mappings are checked for obsoleted or secondary UniProtKB accessions and are updated accordingly. In the automatic process, the manually curated canonical accession is then expanded to include all its isoforms, and sequence alignment is computed for each PDB-UniProtKB pair. Taking only the PDB-UniProtKB pairs with the same source organism or atleast having a common ancestor within one or two levels up to species level in the taxonomy tree and having at least 90% sequence identity, the pair with the highest sequence identity is annotated as the best mapping. Once we have established the mapping between UniProtKB and PDB protein residues, the cross-references from other resources such as Pfam^14^, InterPro^15^, SCOP^16^, CATH^17^, IntEnz^18^, GO^19,20^, Ensembl^21^ and Homologene^23^ are added. The SIFTS annotations are stored in the SIFTS database, which is used to make the data accessible via the PDBe REST API. Individual XML files for each PDB entry with residue-level information are exported and the summary files are generated in CSV/TSV formats. An additional process was designed that reads the data from the SIFTS database and augments the PDB structure files with UniProtKB numbering and structure (SCOP2, and CATH resource) and sequence (Pfam resource) domain annotations. This update yields more consistent, standardised metadata. It is important to note that none of the core PDB information, such as atomic coordinates and experimental data, are altered in any way. Figure 1 shows the schematic overview of the data flow of the SIFTS process and highlights the additional process that was developed to export these data into PDBx/mmCIF files. The process helps researchers and data services access SIFTS data directly from the PDBx/mmCIF^37^ files. To facilitate this update, additional “SIFTS-specific” mmCIF data categories were designed and integrated into the core PDBx/mmCIF data dictionary. These format specifications are discussed in detail below.

### Extensions to the PDBx/mmCIF framework

**Fig. 2:**
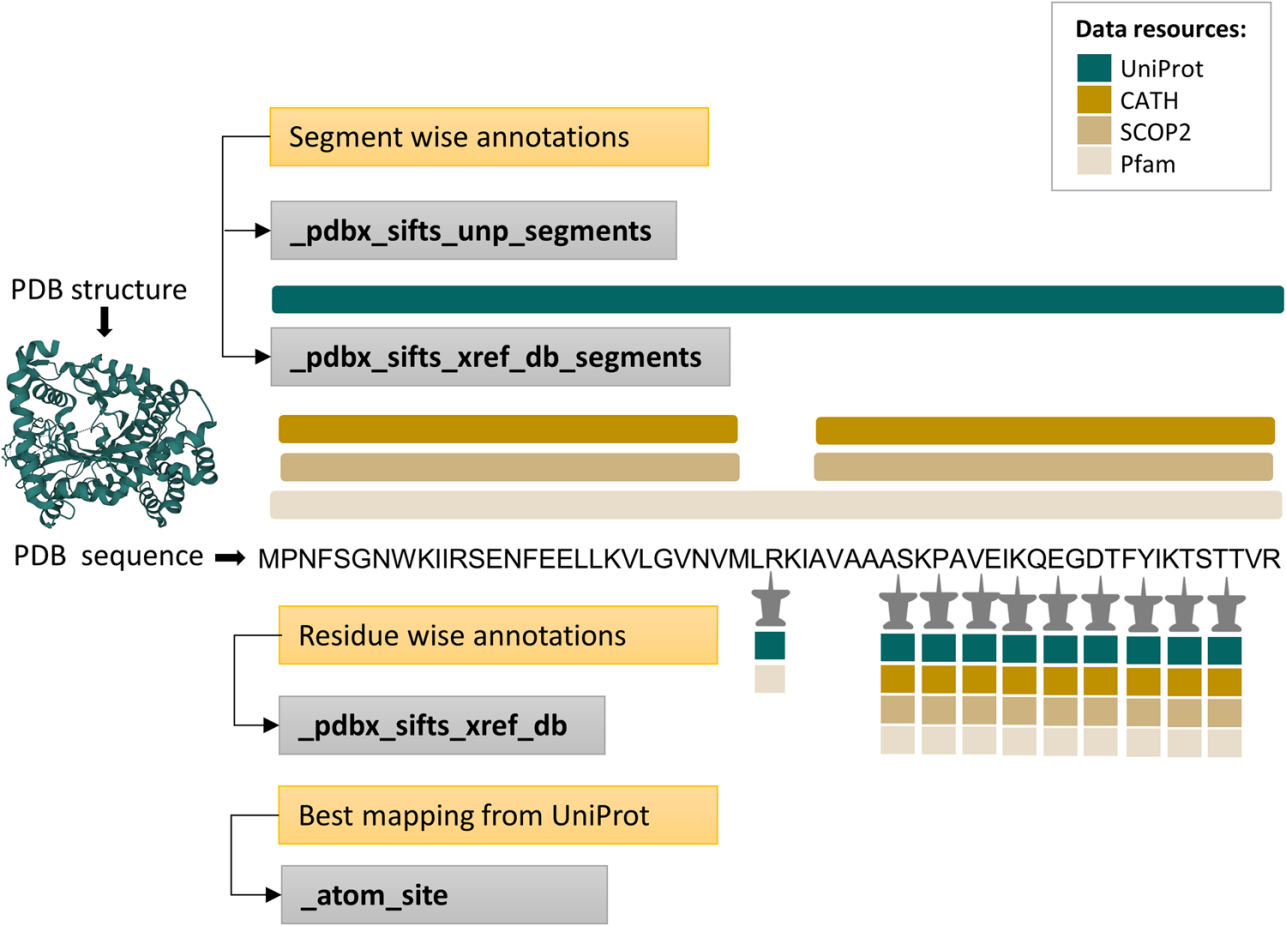
The PDBx/mmCIF extension incorporates mappings from various data resources. SIFTS annotations mapping PDB residues to various data resources are shown both per-segment (top) and per-residue (bottom). All the new SIFTS-specific or modified PDBx/mmCIF categories are shown in grey boxes. The new SIFTS-specific PDBx/mmCIF categories introduced to show per-segment annotations from UniProtKB and all the other external data resources (Pfam, SCOP2, CATH) are “_pdbx_sifts_unp_segments” and “_pdbx_sifts_xref_db_segments” respectively. “_pdbx_sifts_xref_db” is another new SIFTS-specific PDBx/mmCIF category introduced to show per-residue annotations. We also modified the “_atom_site” category to indicate the best mapped UniProtKB sequence.

PDBx/mmCIF framework organises information in categories containing related data items^37^. The updated PDBx/mmCIF files contain the residue mappings between UniProtKB and PDB, and annotations from Pfam, SCOP2, and CATH. The SIFTS annotations are integrated in two ways: per-segment and per-residue. The per-segment annotations refer to a continuous segment in the protein sequence, where only the start and end positions for the annotations are provided. On the other hand, the per-residue annotations expand the segment boundaries to provide annotations for every residue that spans that region. The reason for having both types of annotation is that expanding segment annotations to the residue level can be complex due to factors such as missing residues, insertions, expression tags, and linker regions in the protein sequence. Moreover, the PDB residue numbers are not always uniquely defined and can have insert codes which together with the PDB residue number uniquely identify a particular residue. These factors can lead to gaps in the numbering between residues, which can make it challenging to expand segment annotations to the residue level. Therefore providing both per-segment and per-residue annotations affords the flexibility to visualise and analyse these data in a way that best suits the user needs. New data categories were added to represent these additional per-segment and per-residue mappings (Fig. 2). Two new categories “_pdbx_sifts_unp_segments” and “_pdbx_sifts_xref_db_segments” were added to represent per-segment mapping to UniProtKB and other data resources - Pfam, SCOP2, CATH. A third category, “_pdbx_sifts_xref_db”, was added to provide per-residue mapping from all the external resources. The “_atom_site” category, which represents the coordinate information, was extended with additional data items to integrate UniProtKB residue numbering from the best mapping adjacent to the atomic coordinates.

**Fig. 3:**
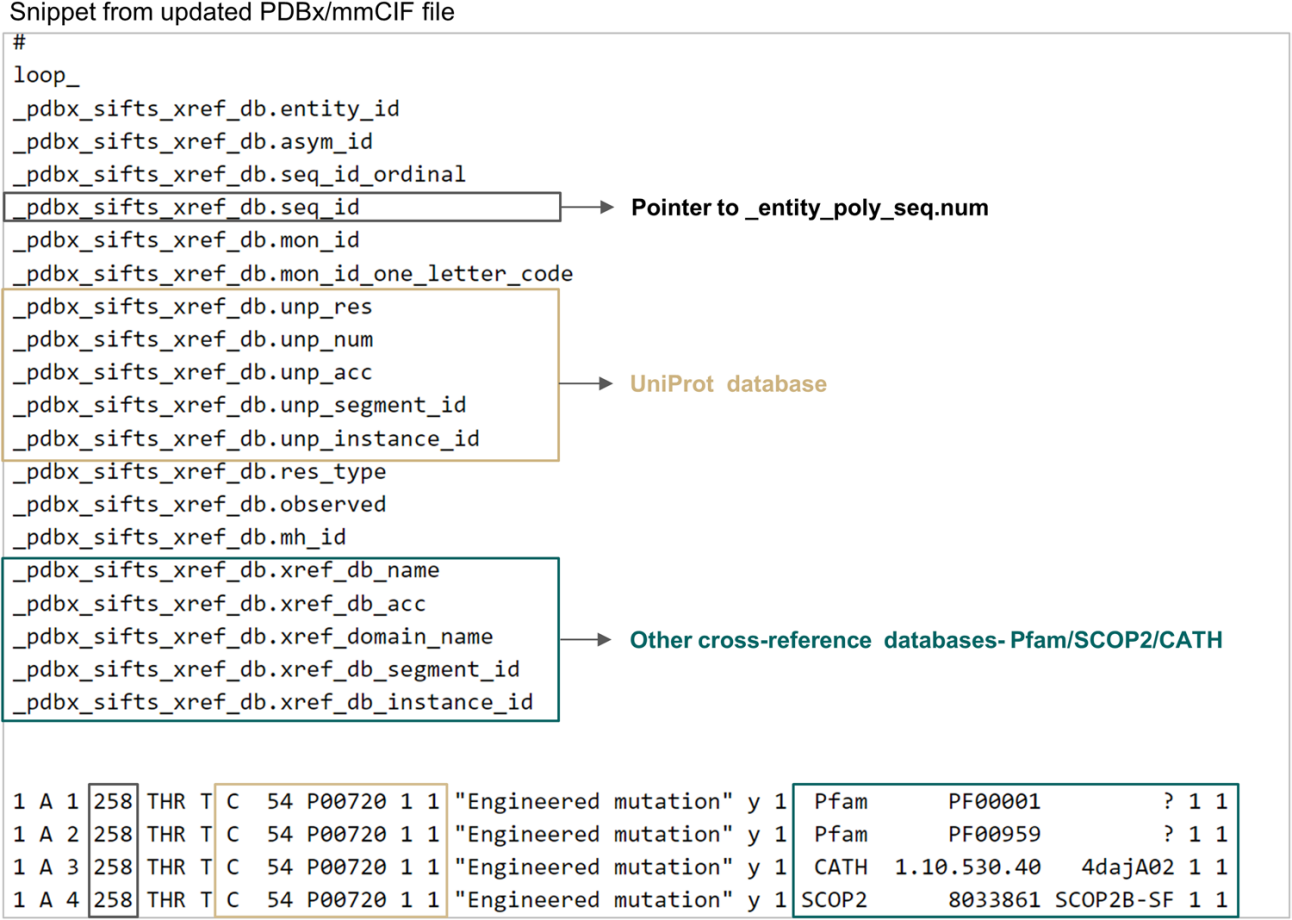
Single placeholder in PDBx/mmCIF files to find all the annotations associated with any residue from external databases. This figure shows the “_pdbx_sifts_xref_db” category for PDB 4daj. This critical new data category can describe residue-level cross-references to external databases. The items specific to the UniProtKB database and other cross-reference databases are marked in beige and green coloured boxes respectively.

A summary of the new and modified data categories necessary to encode the SIFTS annotations data is provided below:

1. **_pdbx_sifts_unp_segments**
  This new category describes residue range-based cross-references specific to the UniProtKB database. It shows segments/regions of PDB residues mapped to the canonical UniProtKB accession and all its isoforms. The residue mapping is established by aligning the PDB sequence to each UniProtKB accession (canonical and all the isoforms) and the sequence identity between the aligned PDB-UniProtKB pair is provided. This category also indicates the best mapped UniProtKB accession.
2. **_pdbx_sifts_xref_db_segments**
  This new category describes residue range-based cross-references to additional databases such as Pfam, SCOP2, and CATH.
3. **_pdbx_sifts_xref_db**
  PDB structures often have missing residues, expression tags or linker regions, making the expansion of mappings from segments (residue range) to individual residues cumbersome. An essential category, “_pdbx_sifts_xref_db”, therefore describes residue level cross-references to external databases. This category provides annotations specific to the best mapped UniProtKB accession and can be used to identify all the mappings for each residue to external databases (Fig. 3).
4. **_atom_site**
  New data items were added to the “_atom_site” category to represent the best mapped UniProtKB accession, residue type and number. The new data item “_atom_site.pdbx_label_index” along with the “atom_site.label_asym_id” provide a unique identifier for all the polymer residues and individual non-polymer and solvent components.

**Fig. 4:**
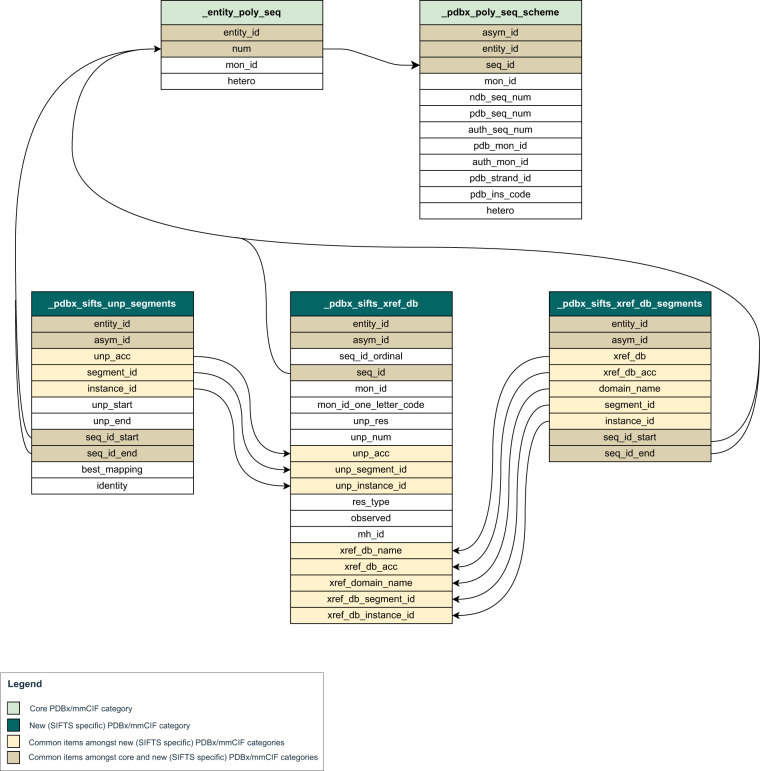
Category relationship diagram including new SIFTS specific PDBx/mmCIF categories. New SIFTS specific PDBx/mmCIF data categories are shown along with their data items. All the common data items amongst these new data categories are highlighted and their relationship is shown. Further, the relationship of the data items representing PDB residue numbers - “.seq_id”, “.seq_id_start” or “.seq_id_end” in these new data categories to existing data categories is shown.

There are two different numbering schemes followed to indicate each residue (amino-acid or nucleotide) in the PDBx/mmCIF file. Firstly, “auth_seq_id” which is the numbering provided by the author. An author can assign its value in any desired way and the values may be used to relate the given structure to a numbering scheme in a homologous structure, including sequence gaps or insertion codes, which are not necessarily numbers. Secondly, “label_seq_id “ which is the wwPDB assigned numbering which starts from 1 and increments sequentially only for all the polymer residues. All the SIFTS-specific categories refer consistently to the wwPDB assigned numbering scheme defined by the “label_seq_id” data item in the atom_site category. The reference to labl_seq_id is provided by the data items “.seq_id”, “.seq_id_start” and “.seq_id_end” in the relevant categories. Data on the author provided or the PDB numbering scheme can be retrieved using the appropriate relationships defined in the PDBx/mmCIF categories (Fig. 4).

**Fig. 5:**
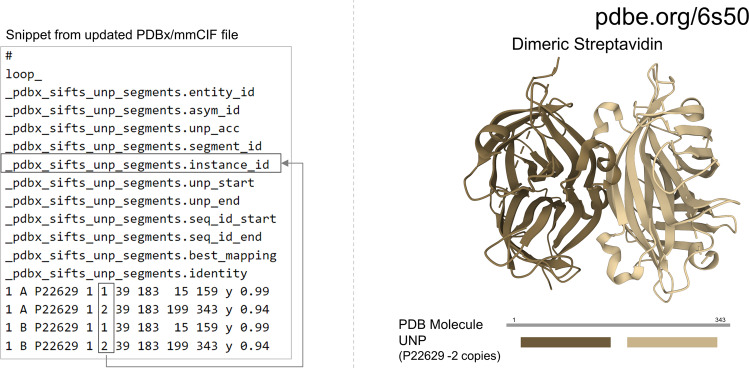
Distinguishing between multiple instances of the same protein in the PDBx/mmCIF file. The data item “.instance_id” enables users to identify the two copies of the same protein, Streptavidin (UniProtKB accession P22629), in the dimeric Streptavidin structure (PDB 6s50).

Often in many proteins, several domains are tandemly repeated^38^. Additionally, researchers also synthesise structures where even the entire protein is repeated for specific research purposes^39,40^. Previously, there was no automated way to find corresponding UniProtKB mappings for multiple domains in a protein structure in the PDB. The data item “.instance_id” is designed to help identify multiple instances of the same protein segment. For example, in the single-chain dimeric Streptavidin structure (PDB 6s50), the two copies of Streptavidin^41^ are easily identified by instance ids “1” and “2” for the UniProtKB accession P22629 (Fig. 5).

Similarly, users can rely on this data item to easily identify multiple copies of the same domains in a protein structure.

**Fig. 6:**
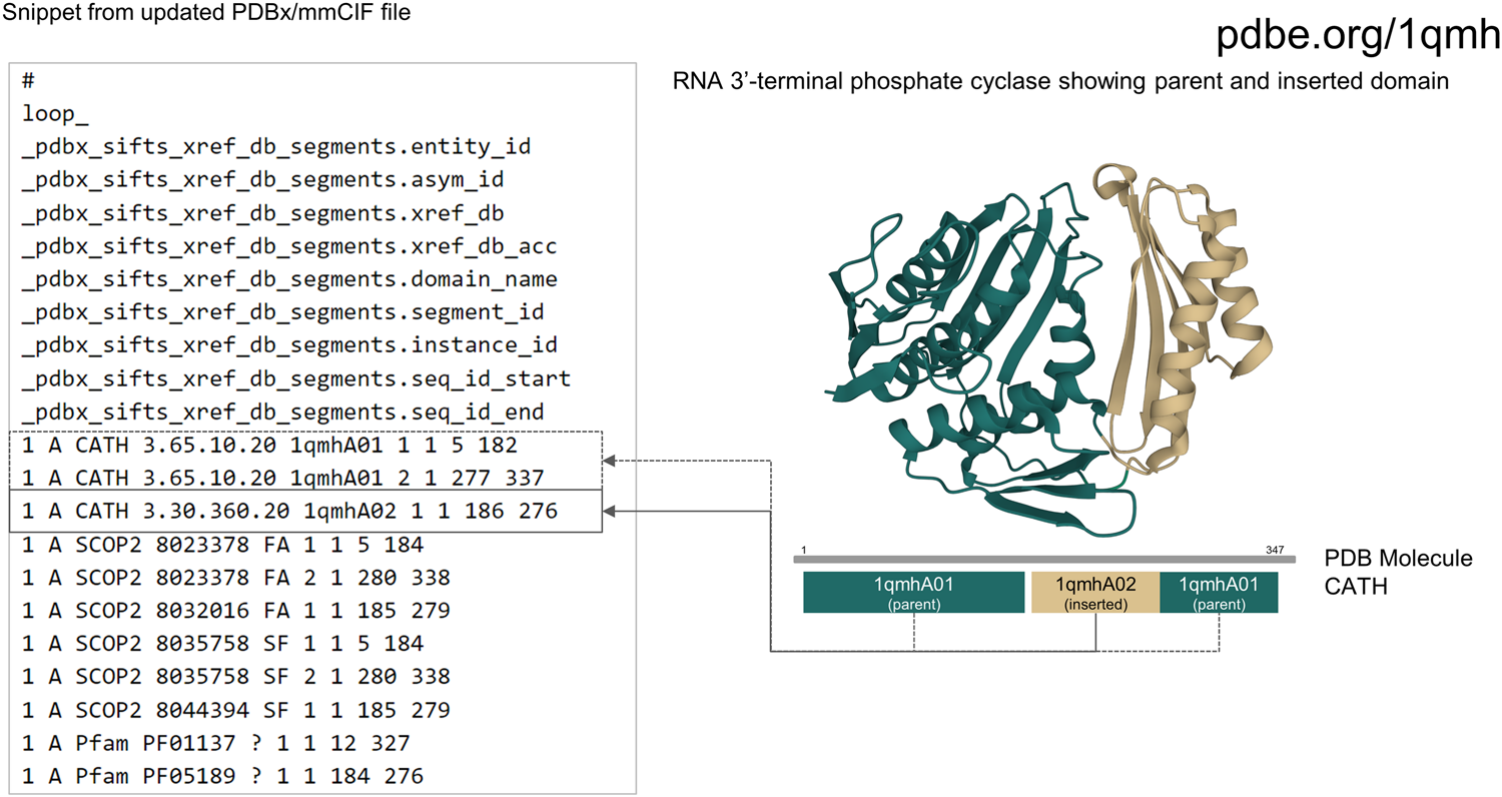
Identification of split domains from PDBx/mmCIF file. The “_pdbx_sifts_xref_db_segments” category in the PDBx/mmCIF file of PDB 4daj helps to clearly identify discontinuous domains. The two halves of the M3 receptor domain are indicated by the same “.instance_id” but different “.segment_id”.

During evolution protein structures may evolve with an insertion of an additional domain which splits the original structural domain into a discontinuous range of residues in the sequence^42^. For example, the *E.coli* enzyme RNA 3′-terminal phosphate cyclase (PDB 1qmh) consists of two structural domains where a smaller insert domain (residues 186–276) splits the larger domain (residues 5–182 and 277–337)^43^. The identification of the split domain (residues 5–182, 277–337) is evident from the “.segment_id” data item (Fig. 6).

Complete documentation for all the new and updated data categories and items is available at https://mmcif.wwpdb.org/dictionaries/ascii/mmcif_pdbx_v50.dic.

### Applications

The SIFTS resource has been widely used in various research studies to retrieve residue correspondence between PDB structures and UniProtKB sequences^44–48^. However, in many cases, researchers have had to manually renumber the coordinate files to reflect UniProtKB numbering for subsequent comparative analysis across multiple PDB structures^49–51^. While SIFTS has been used in several functional studies, including mapping somatic mutations to protein structures to identify 3D clusters of mutations with functional significance^52^ and mapping GPCR structures to their respective G protein structures to investigate the allosteric mechanism of GPCR activation^53^, authors still had to manually validate missing positions in PDB structures to verify genuine cases of chimeric proteins, peptide tags, or point mutations. Unfortunately, this process was both time-consuming and error-prone. However, with the incorporation of SIFTS residue-level mapping to the best mapped UniProtKB sequence in the PDBx/mmCIF files, manual verification is no longer necessary, saving time and facilitating the analysis and interpretation of data.

Integration of UniProtKB sequence annotations and 3D-structures, can furnish the biological and functional context for the structural data. For instance, mapping variant annotations onto 3D-structure, can provide insights into the genetic basis of complex traits and diseases. SIFTS resource has also been used to fetch annotations like sequence domains and structural domains for various PDB structures^51,54^. Using the domain annotations mapped to a protein sequence in these PDBx/mmCIF files, researchers can easily identify the location, multiple copies and boundaries of different domains within a protein, which can help in understanding the overall structure and function of the protein. This also facilitates comparing proteins with similar domain structures and identifies potential functional relationships.

SIFTS is not only widely used in scientific research but also by several data resources^12^. For example, UniProtKB exploits SIFTS information to provide structure mapping in the UniProtKB database. SCOP^55^ and Pfam^14,56^ also use SIFTS to map protein domains and connect sequence domains with their corresponding structures. The web resource Kincore relies on SIFTS to map protein kinases to their respective structures, extract relevant information such as domain boundaries and ligand binding sites, and provide a structural classification of protein kinases and their inhibitors^50^. The PDBx/mmCIF files with SIFTS annotations address the fundamental need by combining data from various resources and providing coordinate files with a common reference frame, improving interoperability and reuse of these data. The availability of these files will streamline data extraction and promote consistent and efficient data sharing.

Adding UniProtKB, Pfam, SCOP2, and CATH annotations to PDB coordinate files can be very helpful for resources like Gene Integration with Function, Taxonomy, and Sequence (GIFTS, https://www.ebi.ac.uk/gifts/), Venus^57^ or PhyreRisk^58^. These annotations provide valuable information to gain a deeper understanding of the relationships between protein structure and function^59^, which can be used to link structural and functional data on a genome-wide scale^60^. By integrating these annotations in PDBx/mmCIF files, it becomes easier to map genetic variants to protein structures, which can greatly facilitate genome-wide studies. The use of SIFTS annotations in the COSMIC data resource is an excellent example of how this approach can be used to efficiently and accurately analyse the impact of genetic variants on protein function and stability^61^. This can be further expanded to support a wide range of computational approaches for analysing protein structure and function^62^, including functional annotation^48^, structural comparison^59^, ligand binding analysis^63^, identifying new protein-protein interactions^64^, functional pathways, and potential drug targets^65^ on a large scale.

Various data visualisation tools can directly use these PDBx/mmCIF files, making the mapping of 1D sequence data onto the 3D structure views straightforward. With our improvements, researchers from various scientific fields can easily map sequence feature data onto PDB structures. Users can directly retrieve all the SIFTS annotations like structural domains, sequence domains and conflicts between sequences and structures from the PDBx/mmCIF files.

**Fig. 7:**
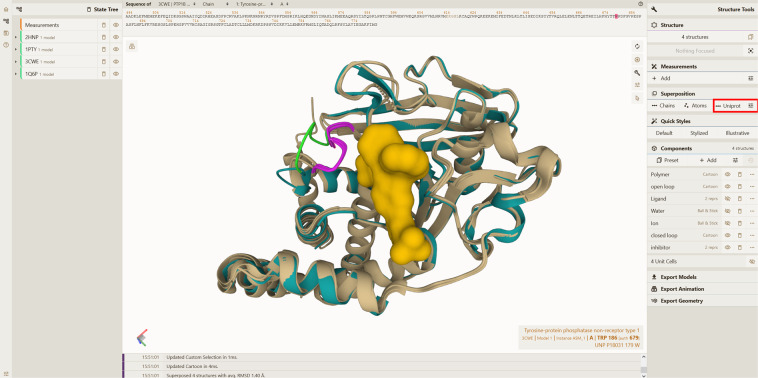
Superposition of protein structures using Mol*. The superposed apo and holo forms of human PTP1B protein are shown in green and beige colours, respectively, in Mol*. The WDP loop is in open (light green colour) conformation in the apo form (PDB 2HNP). Upon binding to various substrates/inhibitors this WDP loop attains closed (pink colour) conformation covering the catalytic site. The inhibitor bound in PDB 1Q6P is shown in the surface representation. The average RMSD between the four superposed structures as computed by Mol* is 1.40 Å. As seen in the tool-tip (bottom-right in the figure), residue W179 from PDB 3CWE and other residues in inhibitor bound PDBs 3CWE and 1Q6P have different author numbering compared to the unbound/substrate bound form (PDB 2HNP/1PTY). The UniProtKB numbering in the PDBx/mmCIF file provides a common reference frame for residue correspondence and supports superposition based on UniProtKB in Mol*.

These files also provide a basis for improved comparisons between experimentally determined and predicted protein models. UniProtKB numbering in the coordinate files allows direct residue correspondence making structural comparison and superposition easier. It also makes it easier to compare PDB structures with the predicted model structures from AlphaFold DB^33,34^, SWISS-MODEL^32^, RoseTTAFold^66^, and many other resources, as these models follow a natural sequence numbering. These files are already being used by Mol*^67^ (https://molstar.org/viewer/) to perform extremely fast superpositions using the SIFTS UniProtKB mapping. This superposition functionality in Mol*^67^ is very powerful as it gives users the means to directly superimpose protein structures in their web browser without downloading any data or software. Mol* uses the SIFTS specific new data items added in the “_atom_site” category to establish the residue equivalence (UniProtKB residue number) from different PDB structures. Mol* superimposes the structures by calculating the optimal rotation and translation that align the corresponding atoms in each equivalent protein residue. Figure 7 shows the superposition of the unbound and bound forms of human Protein Tyrosine Phosphatase 1B protein (PTP1B, UniProtKB accession: P18031) performed using the “UniProt” button (highlighted in red box) in the Mol* Superposition panel^67^. This protein is known to be a signalling molecule regulating a variety of cellular processes including cell growth, differentiation and oncogenic transformation and is a potential therapeutic target for the treatment of type 2 diabetes and cancer^68^. Upon substrate/inhibitor binding, the WPD loop transitions from an open to a closed conformation^69–72^ as shown in Fig. 7.

**Fig. 8:**
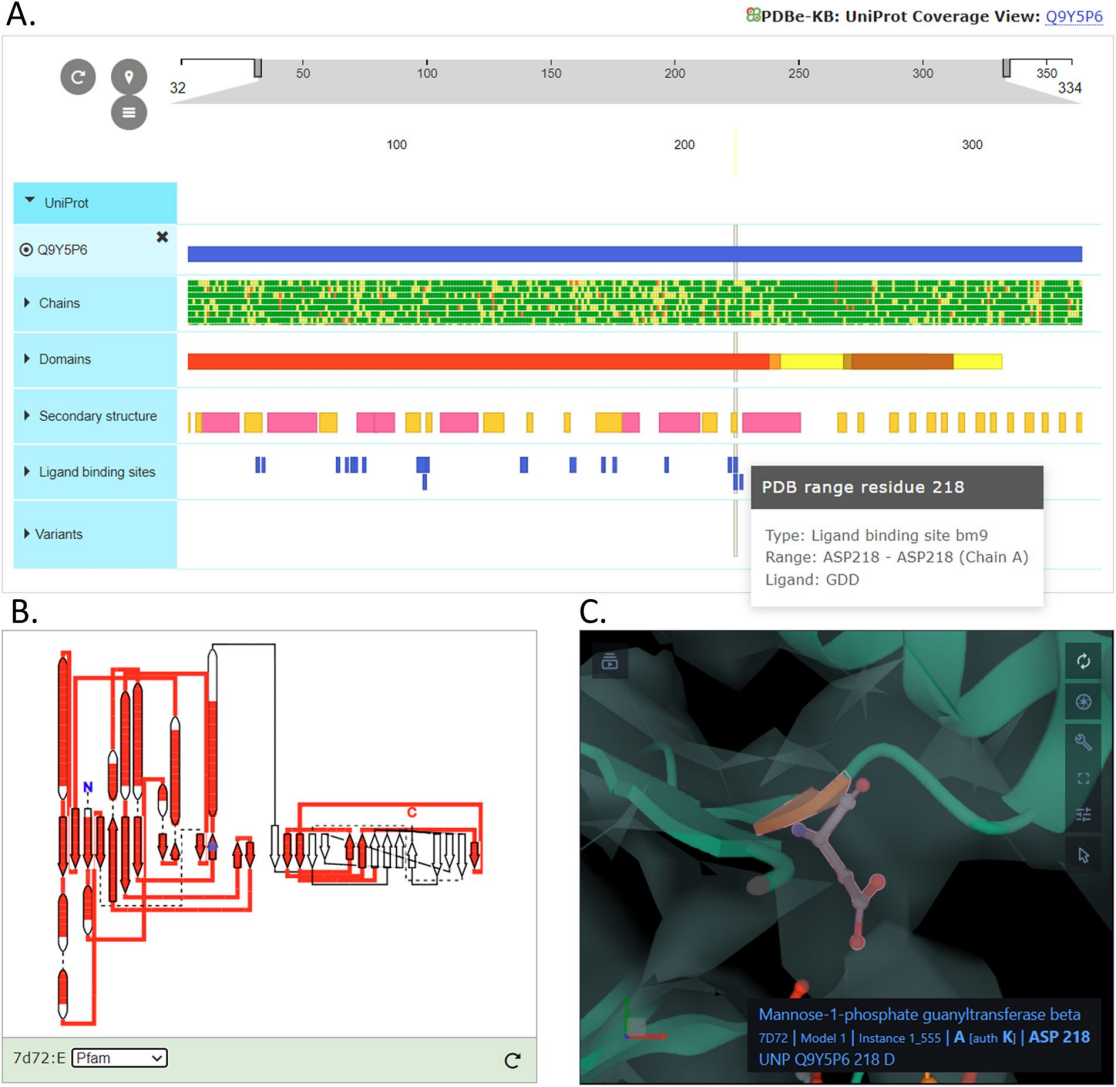
The 2D visualisation components are interactively linked with 3D visualisation components on PDBe entry pages. Various 2D and 3D visualisation components seen on PDBe entry pages are interactively linked with each other. Here we show visualisation data for Mannose-1-phosphate guanyltransferase (PDB 7d72). (**A**) shows a 2D sequence feature viewer (ProtVista) and (**B**) shows a 2D topology viewer, along with (**C**) showing the 3D viewer, Mol*. As users select any residue (here ligand-binding residue ASP218 is selected) in ProtVista, it is automatically highlighted in Mol* and vice-versa. Users can also highlight a range of residues (e.g. domains) in any of these viewers. Here, we show the Pfam domain highlighted in red in the 2D topology viewer.

The new PDBx/mmCIF files also provided a basis for developing interactive visualisations. For example, the PDBe entry pages show the ProtVista component^73^, a 2D visualisation for displaying the primary sequence features of proteins. ProtVista was developed in collaboration with UniProtKB and InterPro at EMBL-EBI. The PDBx/mmCIF files with PDB-UniprotKB residue mapping, enable interactivity between the 3D viewer (Fig. 8C), the ProtVista sequence viewer (Fig. 8A) and the 2D topology component (Fig. 8B). Consequently, Mol* can easily display all the annotations available in ProtVista and the 2D topology component on the 3D structure. As shown in Fig. 8, for Mannose-1-phosphate guanyltransferase, PDB 7d72 (https://www.ebi.ac.uk/pdbe/entry/pdb/7d72/protein/1), if users click on any residue annotation in the 2D viewer ProtVista, the residue or the residue segment is automatically highlighted in 3D,in the Mol* viewer. Similarly, users can highlight various structural or sequence domains, or other annotations in either the 2D topology component, 2D ProtVista component or Mol* viewer, and the three visualisations cross-talk with each other simultaneously, making visualisation and interpretation of data much easier. Mol* already uses these PDBx/mmCIF files to display various annotations on PDBe and PDBe-KB webpages. With SIFTS annotations directly available in the coordinate file, the 3D visualisation on PDBe and PDBe-KB webpages is more efficient and optimal.

It is important to note that adding additional data to a PDBx/mmCIF file, such as augmenting best mapped UniProtKB residue mapping in the “atom_site” category can come with a trade-off of an increase in the file size. While this may not be an issue for smaller PDB entries, it can become problematic for larger entries with significant file size. To address this issue, wwPDB provides binaryCIF^74^ (bcif) files as an alternative to traditional PDBx/mmCIF files. The bcif format is a compressed binary version of the PDBx/mmCIF format that significantly reduces the file size, making it easier to handle and share large amounts of structural data. The Mol*, an open-source software for 3D molecular visualisation and analysis, also supports the bcif file format, allowing users to easily access and analyse structural data in this format.

## Discussion

Interoperability challenges between the protein structure data in the PDB and protein sequences in the UniProtKB presents a significant barrier to accessibility and reusability. The seemingly trivial task of mapping residue-level information proved to be a formidable task that necessitated the development of the SIFTS resource. While SIFTS has successfully provided up-to-date mappings between the PDB and other data resources for the past 20 years, using these mappings still required some level of technical expertise.

To remove a tedious but previously mandatory step in many structural data analyses, we worked on adding the SIFTS mapping data directly into the PDBx/mmCIF files, the master format for the PDB archive. We designed new data categories and extended existing ones to provide flexible support for residue-level annotations. This development will allow easy linking of structural and functional annotations derived using structure and sequence data. It will also streamline the vast majority of high-throughput bioinformatics analysis pipelines by allowing developers to remove a tedious and error-prone step from their processes. Including the SIFTS data in the PDBx/mmCIF will also improve the efficiency of data visualisation tools, both those that specialise in 3D molecular graphics and those that focus on the interactive mapping of annotations onto to the protein structure representations e.g. sequence or topology.

By extending the PDBx/mmCIF data format, this work has laid the foundation for the future integration of additional annotations, allowing the files to be more comprehensive and to provide the biological context for PDB structures.

## Methods

### PDBx/mmCIF file format and PDBx/mmCIF dictionary

The PDBx/mmCIF(Protein Data Bank exchange/macromolecular Crystallographic Information File) is a well-established data format utilised for storing and sharing information related to the three-dimensional structure of macromolecules, including proteins and nucleic acids. Widely considered as the master format for the PDB archive, it is extensively used for representing structural data. The format uses a text-based file format that encodes data and metadata utilising data items grouped into categories. The PDBx/mmCIF dictionary^30^ defines a standardised set of categories and data items, along with controlled vocabularies and explicit relationships between different categories and data items. This format is extensible, allowing the incorporation of new data items and categories, as demonstrated by the IHM^75^ and ModelCIF^76^ extensions. The IHM extension enables the archiving of structural models of macromolecular assemblies obtained through integrative/hybrid methods, while the ModelCIF extension enables the consistent representation of molecular models obtained through computational methods. By facilitating such inclusion of new information and accommodating scientific advancements, the PDBx/mmCIF dictionary continues to remain relevant and valuable to the scientific community. The PDBx/mmCIF dictionary is maintained by the wwPDB consortium and is regularly updated with new data items to reflect changes in the field of structural biology. The mmCIF dictionary can be accessed and downloaded freely from https://mmcif.wwpdb.org/dictionaries/mmcif_pdbx_v50.dic/Index/.

### SIFTS-specific data categories and items in PDBx/mmCIF Dictionary

The PDBx/mmCIF dictionary was extended with, three new data categories to provide the necessary semantic organisation to represent SIFTS annotations: “_pdbx_sifts_unp_segments”, “_pdbx_sifts_xref_db_segments”, and “_pdbx_sifts_xref_db”.

The “_pdbx_sifts_unp_segments” category displays the UniProtKB sequence segments that correspond to the PDB structure. The “_pdbx_sifts_xref_db_segments” category provides information about the cross-references between the PDB structure and other databases, such as Pfam, CATH, and SCOP2. Finally, the “_pdbx_sifts_xref_db” category displays per-residue annotations between the PDB structure, UniProtKB, and other data resources.

Additionally, the “_atom_site” category was modified to integrate residue-level cross-reference data to the best mapped UniProtKB sequence. The updated PDBx/mmCIF dictionary, including all the new and updated data categories and items, is publicly available at https://mmcif.wwpdb.org/dictionaries/mmcif_pdbx_v50.dic/Index/.

### Augmenting the core SIFTS process

SIFTS (Structure Integration with Function, Taxonomy and Sequences) is a collaborative resource between the PDBe (Protein Data Bank in Europe) and UniProtKB teams at EMBL-EBI. It is designed to map the protein structures available in PDB to the protein sequences in UniProtKB at the individual residue level. The SIFTS mapping can facilitate transfer annotations from a variety of biological resources including the NCBI taxonomy database, IntEnz, GO, Pfam, InterPro, SCOP, CATH, PubMed, Ensembl, Homologene, and automatic Pfam domain assignments based on HMM profiles. The pipeline is run weekly by PDBe as part of the PDB release process.

The mapping between PDB protein structures and UniProtKB protein sequences is manually curated by PDB and UniProtKB annotators. SIFTS performs automatic sequence alignment and generates a residue-level mapping between aligned protein structures and sequences. The pipeline downloads and parses data from various biological resources, which is then loaded into the SIFTS database (Fig. 1). SIFTS database is queried to derive residue-level annotations for all these biological resources. The SIFTS process generates per-entry XML files, summary CSV and TSV files to distribute all the SIFTS annotations. The SIFTS database also powers all the SIFTS related PDBe API^29^.

To update PDBx/mmCIF files with residue-level annotations from SIFTS resources, a new process was added to the existing SIFTS pipeline. For a given PDB entry, the new process reads all the relevant data from the SIFTS database and integrates it into the PDBx/mmCIF file. The integration of SIFTS data uses the extended PDBx/mmCIF dictionary discussed earlier. The new process is implemented in Python and uses gemmi^77^ to parse the PDBx/mmCIF file and write the SIFTS annotations in the corresponding categories. The process is executed as part of the PDBe weekly release pipeline, ensuring up-to-date SIFTS data in the PDBx/mmCIF files every Wednesday to coincide with the weekly PDB release. Currently, residue-level SIFTS annotations for UniProtKB, Pfam, SCOP2, and CATH databases are integrated in the PDBx/mmCIF files.

## Data Availability

We expanded the PDBe release pipeline with a process that adds SIFTS annotations to the PDBx/mmCIF files for individual structures in the PDB archive. The scientific community can download these PDBx/mmCIF files from the PDBe entry pages (https://pdbe.org/7dr0) and through direct URLs (https://www.ebi.ac.uk/pdbe/static/entry/7o9f_updated.cif), using the PDBe download service (https://www.ebi.ac.uk/pdbe/download/api) or from the EMBL-EBI FTP area (https://ftp.ebi.ac.uk/pub/databases/msd/updated_mmcif/).

## Code availability

To assist users in utilising the updated PDBx/mmCIF files and SIFTS annotations, a Google Colab notebook is available at https://colab.research.google.com/github/PDBe-KB/sifts_data_analysis/blob/main/sifts.ipynb or via GitHub at https://github.com/PDBe-KB/sifts_data_analysis. This notebook provides information on how to parse, extract and filter SIFTS annotations from the updated PDBx/mmCIF files. Additionally, the notebook demonstrates how users can compare various numbering schemes of a given residue across different PDB structures of the same protein.
